# In vitro and in vivo comparison of the anti-staphylococcal efficacy of generic products and the innovator of oxacillin

**DOI:** 10.1186/1471-2334-10-153

**Published:** 2010-06-04

**Authors:** Carlos A Rodriguez, Maria Agudelo, Andres F Zuluaga, Omar Vesga

**Affiliations:** 1 Professor, Department of Pharmacology & Toxicology, University of Antioquia Medical School, Calle 62 # 52-59, Lab. 630 Medellín, Colombia; 2 Professor of Medicine, Section of Infectious Diseases, Department of Internal Medicine, University of Antioquia Medical School, Calle 62 # 52-59, Lab. 630 Medellín, Colombia; 3 GRIPE (Grupo Investigador de Problemas en Enfermedades Infecciosas), University of Antioquia Medical School, Calle 62 # 52-59, Lab. 630 Medellín, Colombia

## Abstract

**Background:**

Oxacillin continues to be an important agent in the treatment of staphylococcal infections; many generic products are available and the only requirement for their approval is demonstration of pharmaceutical equivalence. We tested the assumption that pharmaceutical equivalence predicts therapeutic equivalence by comparing 11 generics with the innovator product in terms of concentration of the active pharmaceutical ingredient (API), minimal inhibitory (MIC) and bactericidal concentrations (MBC), and antibacterial efficacy in the neutropenic mouse thigh infection model.

**Methods:**

The API in each product was measured by a validated microbiological assay and compared by slope (potency) and intercept (concentration) analysis of linear regressions. MIC and MBC were determined by broth microdilution according to Clinical and Laboratory Standard Institute (CLSI) guidelines. For in vivo efficacy, neutropenic ICR mice were inoculated with a clinical strain of *Staphylococcus aureus*. The animals had 4.14 ± 0.18 log_10 _CFU/thigh when treatment started. Groups of 10 mice per product received a total dose ranging from 2.93 to 750 mg/kg per day administered q1h. Sigmoidal dose-response curves were generated by nonlinear regression fitted to Hill equation to compute maximum effect (E_max_), slope (N), and the effective dose reaching 50% of the E_max _(ED_50_). Based on these results, bacteriostatic dose (BD) and dose needed to kill the first log of bacteria (1LKD) were also determined.

**Results:**

4 generic products failed pharmaceutical equivalence due to significant differences in potency; however, all products were undistinguishable from the innovator in terms of MIC and MBC. Independently of their status with respect to pharmaceutical equivalence or in vitro activity, all generics failed therapeutic equivalence in vivo, displaying significantly lower E_max _and requiring greater BD and 1LKD, or fitting to a non-sigmoidal model.

**Conclusions:**

Pharmaceutical or in vitro equivalence did not entail therapeutic equivalence for oxacillin generic products, indicating that criteria for approval deserve review to include evaluation of in vivo efficacy.

## Background

Penicillinase-resistant penicillins, including the isoxazolyl penicillin oxacillin (OXA), have been the mainstay treatment of β-lactamase producing *Staphylococcus aureus *infections since the 1960s, although their usefulness is nowadays reduced by the emergence and worldwide dissemination of methicillin-resistant strains (MRSA) [1]. The patents of these drugs expired long ago and many generic products are currently available while the innovator has abandoned production of this essential antibiotic. The only requirement by drug regulatory agencies (DRA) to authorize marketing of generic intravenous drugs is the demonstration of pharmaceutical equivalence, defined as containing identical amounts of the same active ingredients in the same dosage form and manufactured in compliance with current Good Manufacturing Practices guidelines. Bioequivalence tests are waived, as bioavailability of intravenous formulations is by definition 100% [2-4], and therapeutic equivalence (defined as having the same efficacy and safety profile of the comparator) is assumed from pharmaceutical equivalence without further testing.

Our aim was to challenge the assumption that pharmaceutical equivalence is a surrogate predictor of therapeutic equivalence for generic oxacillin, comparing with the innovator product concentration and potency of the active pharmaceutical ingredient (API), in vitro activity, and in vivo efficacy against a clinical strain of *S. aureus *in the neutropenic mouse thigh infection model (NMTIM). Preliminary results of this work were presented at the 44^th ^ICAAC [5].

## Methods

### Bacteria, media and antibiotics

We used the wild type clinical strain *S. aureus *GRP-0057 for all experiments designed to determine antibacterial efficacy; the microorganism was grown to log phase in Mueller-Hinton broth and agar for susceptibility tests and in Trypticase Soy broth and agar for in vivo studies (all from Becton Dickinson, Sparks, MD, USA). We used *S. aureus *ATCC 29213 as quality control strain for susceptibility testing, following recommendations from Clinical and Laboratory Standards Institute (CLSI). *S. aureus *ATCC 6538p was the seeding organism on Difco^® ^Antibiotic Medium 1 for microbiological assays. Antibiotics were bought from well-reputed local drugstores and reconstituted according to manufacturer instructions. The products, all licensed for human use by Invima (the Colombian DRA), included 11 oxacillin generics manufactured in Colombia and the innovator product (Prostafilina^®^, Bristol-Myers Squibb, Mexico). To facilitate data illustration, we use abbreviated names taking the first 3 letters of the manufacturer. Table 1 lists studied products and their code, maker, batches and country of manufacture; the last column describes the tests performed, as not all generic products were available at the time of every experiment.

**Table 1:** Manufacturing information of oxacillin products included in the study (11 generics plus the innovator).

OXACILLIN (OXA) PRODUCTS	BATCH	MANUFACTURER	DISTRIBUTOR	PERFORMED TESTS
**OXA-BMS (innovator)**	EL059 EG187 EF109 EF372 ED259	Bristol-Myers Squibb de Mexico.	Bristol-Myers Squibb	MA, MIC&MBC, NMTIM

**OXA-BLA**	A 4602 B 4603	Vitrofarma SA, Bogota, Colombia	Laboratorios Blaskov	MA, MIC&MBC, NMTIM

**OXA-CAR**	309V1104 378V0705	Farmacologica SA, Bogota, Colombia	Carlon Ltda	MA, MIC&MBC, NMTIM

**OXA-COL**	0010202 05103	Farmacologica SA, Bogota, Colombia	Colpharma Ltda	MA, MIC&MBC, NMTIM

OXA-EXP*	OX 010462	Farmacologica SA Bogota, Colombia	Laboratorios Expopharma	MA, NMTIM

**OXA-MEM**	110291 050239 090281	Vitrofarma SA, Bogota, Colombia	Memphis Products	MA, MIC&MBC, NMTIM

**OXA-OPH**	080267 060249	Vitrofarma SA, Bogota, Colombia	Laboratorios Farmaceuticos Ophalac	MA, MIC&MBC, NMTIM

OXA-PEN*	20275 30417	Farmacologica SA, Bogota, Colombia	Pentacoop SA	MA, MIC&MBC, NMTIM

**OXA-QIM**	6400702	Vitrofarma SA, Bogota, Colombia	Quimicol	MIC&MBC, NMTIM

**OXA-SCA**	OX-010462	Farmacologica SA, Bogota, Colombia	Farmionni Scalpi S.A.	MA, NMTIM

**OXA-SER**	OX-010508	Farmacologica SA Bogota, Colombia	Serpharma SA	MIC&MBC, NMTIM

**OXA-VIT**	120198 090281 110291	Vitrofarma SA, Bogota, Colombia	Vitalis	MA, MIC&MBC, NMTIM

**Abbreviators**: MA: microbiological assay; MIC&MBC: minimal inhibitory concentration and minimal bactericidal concentration; NMTIM: neutropenic mouse thigh infection model. *The data from one experiment involving these two generic products and the innovator had to be excluded from the analysis due to heavy contamination of agar plates containing thigh homogenates that prevented reliable colony counting

### Microbiological assay

The concentration of API was determined by a previously validated assay [6]. The innovator product and 9 generics were tested simultaneously in a 36×36 cm plate originally described by Bennett in 1966 [7]; 2 generics were not available at the time of these assays (OXA-QIM and OXA-SER). Pharmaceutical equivalence was defined as a generic product displaying a regression line parallel and overlaid to that of the innovator (P > 0.05 by CFA). On the other hand, parallel curves with significantly different intercepts indicated the same API but at higher or lower concentration, while lack of parallelism implied differences in potency.

### In vitro activity

Following CLSI methods [8], we determined minimal inhibitory (MIC) and bactericidal (MBC) concentrations by broth microdilution (1-3 assays, each by duplicate) of 9 generic products, and compared them with the innovator (gold standard) by means of the Kruskal-Wallis test. The generic products OXA-EXP and OXA-SCA were not available at the time of these experiments.

### Neutropenic mouse thigh infection model

Eleven generic products were available for in vivo evaluation, however two of them (OXA-EXP and OXA-PEN) had to be excluded from the analysis due to contamination of the plates which altered the reliability of CFU counts. Six-week-old specific-pathogen-free female mice of the strain Udea:ICR(CD-1) weighing 23-27 g were rendered deeply neutropenic (≤10 neutrophils/μL) by intraperitoneal injections of cyclophosphamide (Cytoxan^®^, Bristol-Myers Squibb Co., New Brunswick, NJ, USA) 4 days (150 mg/kg) and 1 day (100 mg/kg) before infection [9]. Sixteen hours after the second cyclophosphamide dose, the animals were inoculated into each thigh with 0.1 mL of a log-phased culture of *S. aureus *GRP-0057 adjusted to 4.0 log_10 _CFU/mL. Groups of 10 mice per product received 5 different total doses ranging from no effect to maximum effect, allocating subgroups of 2 animals to each of 2.93, 11.7, 46.9, 187.5, and 750 mg/kg per day. To optimize the pharmacodynamic (PD) index predicting efficacy for β-lactams (time above MIC), treatment was administered q1h by 0.2 mL subcutaneous injections [10]. Two infected but untreated control mice were sacrificed right after inoculation (-2 h), at the onset (0 h), and at the end of therapy (24 h), when all other (treated) mice were euthanized and their thighs dissected under aseptic technique, homogenized, serially diluted, plated by duplicate on solid medium, and aerobically incubated at 37°C for 18 hours. Data were registered as log_10 _CFU/g and the limit of detection was 2.0 log_10 _CFU/g. To determine net antibacterial effect, the number of CFU remaining in the thighs after 24 hours of treatment was subtracted from the number of CFU that grew in the thighs of control mice during the same period. The experimental protocol was reviewed and approved by the University of Antioquia Animal Experimentation Ethics Committee.

### Statistical analysis of in vivo data

A sigmoid dose-response model described by the Hill equation was used to analyze and determine in vivo efficacy:(1)

where E is the net antibacterial effect (log_10 _CFU/g) after 24 h of treatment, D is the oxacillin dose (mg/kg per day), and E_max _(maximum effect in log_10 _CFU/g), ED_50 _(Effective Dose needed to reach 50% of the E_max_) and N (Hill's slope describing the sensitivity of the dose-effect relationship) are the primary pharmacodynamic parameters (PDP) to be calculated by least-squares nonlinear regression [11]. When possible, we also calculated the secondary PDP bacteriostatic dose (BD) and the dose needed to kill the first log of bacteria (1LKD), both expressed as mg/kg per day (SigmaPlot 10). The number of CFU/g obtained from untreated control mice at 0 h subtracted from that of untreated controls at 24 h represents the net bacterial growth (G) in the absence of therapy, introduced as a constant in the next two equations used to compute secondary PDP:(2)(3)

PDP obtained for each generic product were compared against those of the innovator by the overall test for coincidence of nonlinear regression (Prism 5, GraphPad Software). Accepting a 5% chance for a type I error, the treatment of 10 animals per product to compare 9 generic products with the innovator oxacillin confers 87.3% power to reject the null hypothesis if the magnitude of the difference in antibacterial efficacy is ≥1.0 log_10 _CFU/g and the standard deviation (SD) is <0.5 log_10 _CFU/g. Such difference between generics and the innovator represents a net bactericidal effect greater than 100,000 bacterial cells per gram of tissue, a threshold value several orders of magnitude greater than what would be considered important in clinical medicine.

Instead of the expected sigmoid Hill's pharmacodynamic pattern, one product displayed a U-shaped dose-effect curve. The best fit for such pattern is the Gaussian model, as described by Christopoulos for compounds with simultaneous agonistic and antagonistic actions [11]:(4)

where(5)

Basal represents response in the absence of antibiotic, Range is the maximal inhibitory response value lying within the Basal and the deepest point of the Gaussian curve (E_max _- Basal), and Slope is a fitting constant that describes the particular form of the bell-shaped curve (should not be confused with Hill's slope) [12]. LogED_50 _is the logarithm of the effective dose needed to reach 50% of the E_max_. The expression 10^log[A] ^in Equation 4 corresponds to the logarithmic form in which the dose is introduced in all dose-response relationships: [A] is the independent variable, represented here by the 24 h total dose. Since Basal is zero (CFU/g_controls _- CFU/g_treated _= 0 without treatment), Range equals E_max _in our Gaussian model, therefore(6)

If generic and innovator fit different pharmacodynamic models, they are not therapeutically equivalent by definition: the World Health Organization defines two products as therapeutically equivalent "if they are pharmaceutically equivalent and, after administration in the same molar dose, their effects with respect to both efficacy and safety **are essentially the same**, as determined from appropriate bioequivalence, **pharmacodynamic**, clinical or in vitro studies" (bold by us). To establish which model more appropriately described the dose-effect relationship of each oxacillin product, we ran their respective NLR under both models, and then computed the probability of each model by corrected Akaike's Information Criteria with Prism 5.

In addition, we ran all products simultaneously under Hill's model (multiple NLR, M-NLR) fixing E_max _to the value of the innovator, which permits calculation of hypothetical ED_50 _and *N *values for generic products "forced" to reach the innovator's efficacy, as clinicians try by increasing the dose of generics. The null hypothesis (generic product = innovator product) and our experimental design (simultaneous evaluation of generic and innovator) allow this M-NLR approach which, by giving PDP for all products without violating NLR assumptions, validates an alternative comparison by CFA with all products under Hill model.

## Results

### Microbiological assay

**Figure 1:**
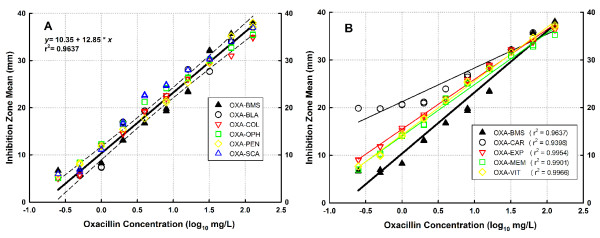
**Concentration-response relationship of innovator and generic products of oxacillin in the microbiological assay**. **A**. The slopes and intercepts of OXA-BLA, OXA-COL, OXA-OPH, OXA-PEN, and OXA-SCA were not statistically different from those of OXA-BMS (innovator), thus confirming their pharmaceutical equivalence (P = 0.1165). The standard curves of all products are better described by a single linear regression, shown here with the 95% confidence interval. **B**. The slopes and intercepts of OXA-CAR, OXA-EXP, OXA-MEM and OXA-VIT were significantly different to the innovator's (P < 0.03458), thus failing pharmaceutical equivalence. As generic products belong to populations different to that of the innovator, each is described by an independent linear regression with their respective coefficient of determination (r^2^).

Panel A of Figure 1 shows the standard curves generated by linear regression of innovator oxacillin and 5 generic products that demonstrated pharmaceutical equivalence (i.e. without significant differences in slope neither intercept with respect to the innovator); all data belong to the same population and therefore are best fitted by a single linear regression. These products were OXA-BLA, OXA-COL, OXA-OPH, OXA-PEN, and OXA-SCA, and their relative potencies ranged between 96.9 and 100.6% with respect to the innovator.

Panel B of Figure 1 displays the regression lines of innovator oxacillin and 4 generics that exhibited statistically significant differences in slope (OXA-CAR, OXA-EXP, OXA-MEM, and OXA-VIT; P < 0.03458), indicating pharmaceutical inequivalence due to alterations in the potency of their API (these 4 generics belong to populations different to that of the innovator product, therefore each is fitted with its own regression). Relative potency of these 4 products was 85.6, 92.4, 95.3, and 94.0%, respectively. OXA-CAR (batch 378v0705) was the only product which did not dissolve completely, in accordance with a grossly different standard curve (Figure 1, panel B). For subsequent experiments with OXA-CAR different ampoules without dissolution problems were used (batch 309v1104).

### In vitro activity

Table 2 presents the MIC, MBC and MBC/MIC ratio of the innovator and 9 generic products of oxacillin against *S. aureus *ATCC 29213 and *S. aureus *GRP-0057.There were no differences with the innovator, including the products lacking pharmaceutical equivalence (see Discussion).

**Table 2:** In vitro activity of 9 generic products of oxacillin and the innovator against two strains of *S. aureus*.

OXACILLIN (OXA) PRODUCTS	*S. aureus *ATCC 29213			*S. aureus *GRP-0057		
	
	MIC (mg/L)	MBC (mg/L)	MBC/MIC Ratio	MIC (mg/L)	MBC (mg/L)	MBC/MIC Ratio
**OXA-BMS (innovator)**	0.22	0.40	1.78	0.16	0.25	1.59

**OXA-BLA**	0.50	0.50	1.00	0.18	0.50	2.83

**OXA-CAR**	0.50	1.00	2.00	0.50	0.50	1.00

**OXA-COL**	0.50	0.71	1.41	0.18	0.35	2.00

**OXA-MEM**	0.13	0.13	1.00	0.13	0.13	1.00

**OXA-OPH**	0.25	0.71	2.83	0.18	0.25	1.41

**OXA-PEN**	0.25	0.35	1.41	0.18	0.21	1.19

**OXA-QIM**	0.50	1.00	2.00	0.18	0.25	1.41

**OXA-SER**	0.25	0.50	2.00	0.25	0.50	2.00

**OXA-VIT**	0.13	0.13	1.00	0.13	0.13	1.00

Exact P (KW*)	0.2115	0.2110	0.2611	0.3650	0.1339	0.2751

Values represent geometric means of each MIC, MBC and MBC/MIC ratio, obtained from 1 to 3 duplicate determinations (ranges not included for brevity and clarity). * Kruskal-Wallis test.

### Neutropenic mouse thigh infection model

**Figure 2:**
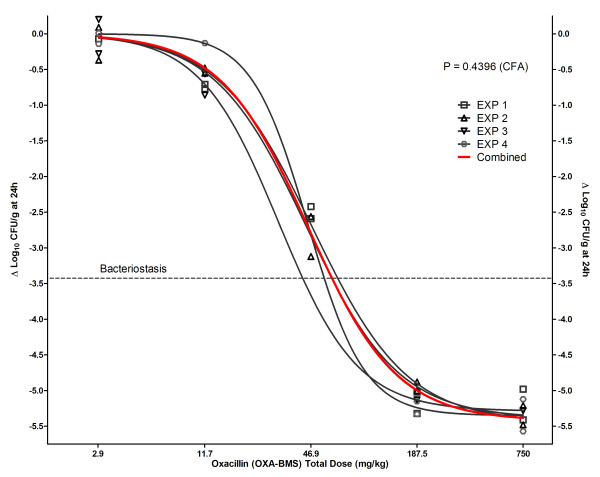
**Dose-response relationship of innovator oxacillin (OXA-BMS) in 4 independent experiments in the neutropenic mouse thigh infection model, fitted to Hill's model by NLR**. The four experiments (gray lines) were indistinguishable by curve fitting analysis (CFA), thus a single regression (red curve) described them better than 4 individual regressions.

A total of 4 technically valid in vivo experiments comparing the innovator with generic products were performed. Table 3 shows the bacterial loads in untreated controls at 0 and 24 h (when oxacillin was started and ended, respectively, in the treatment groups), and the net growths achieved. To test the reproducibility of the animal model, we compared by CFA the 4 dose-response curves obtained from these experiments for the innovator product OXA-BMS (Figure 2), and showed that all data sets were best described by 1 rather than 4 separate regression lines (P = 0.4396), indicating that all data in the innovator's NLR came from the same population. One additional experiment comparing OXA-BMS, OXA-EXP and OXA-PEN was excluded from the analysis because it was heavily contaminated with Gram-negative bacilli that altered *S. aureus *colony counting and generated falsely higher antibacterial effects for all products.

**Table 3:** Bacterial loads and net growths in untreated control mice at the beginning and end of treatment.

EXPERIMENT	**Bacterial Load at 0 h (log**_**10 **_**CFU/g)**		**Bacterial Load at 24 h (log**_**10 **_**CFU/g)**		**Net Growth G (log**_**10 **_**CFU/g)**
		
	Mean	SD	Mean	SD	
1	3.96	0.035	7.86	0.011	3.90

2	4.09	0.106	7.48	0.269	3.39

3	3.85	0.007	7.29	0.347	3.44

4	4.66	0.070	7.57	0.004	2.91

**Mean ± SEM**	**4.14 ± 0.180**		**7.55 ± 0.119**		**3.41 ± 0.202**

**Figure 3:**
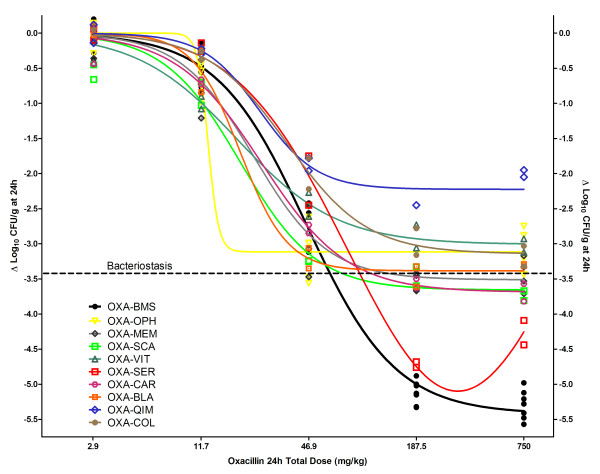
**Dose-response relationship of the innovator and 9 generic products of oxacillin in the neutropenic mouse thigh infection model**. OXA-BMS (innovator, black curve) and 8 generics fitted to Hill's sigmoid model, while generic product OXA-SER fitted to the Gaussian U-shaped model (red curve). Regardless of pharmaceutical equivalence and in vitro activity, all generics displayed significantly inferior bactericidal efficacy (P < 0.0001) or different pharmacodynamic behavior (Gaussian instead of sigmoid) compared with the innovator, thus lacking therapeutic equivalence.

Figure 3 and Table 4 show the dose-response relationships and magnitudes of the PDP obtained by NLR for 9 oxacillin generic products and the innovator from the four combined in vivo experiments. All generics failed to reach the innovator's maximum effect regardless their pharmaceutical equivalence: the most and least effective products killed 22- and 1585-fold fewer microorganisms per gram of tissue respectively than OXA-BMS. In vivo potency of generic products was also significantly inferior: OXA-CAR and OXA-COL required respectively 1.57- and 2.13-fold higher dose for bacteriostasis compared with the innovator (BD = 62.2 mg/kg), other 6 products did not reach enough efficacy to calculate their BD (i.e., their maximum effect was lower than the bacterial growth in control animals), one (OXA-SER) did not fit to Hill's model, and no one killed enough bacteria to calculate their 1LKD (it was 108.4 mg/kg per day for OXA-BMS). Tables 4 and 5 show that in all cases where secondary PDP could not be computed due to lack of efficacy, the only accurate conclusion is that the magnitude of the PDP is greater than the maximum dose used in vivo (750 mg/kg per day).

**Table 4:** In vivo pharmacodynamic parameters of generic and innovator products of oxacillin by nonlinear regression from 4 independent experiments.

OXACILLIN (OXA) PRODUCTS	**E**_**max **_**(log**_**10 **_**CFU/g)**	SEM	N	SEM	**ED**_**50 **_**(mg/kg)**	SEM	BD (mg/kg)	SEM	1 LKD (mg/kg)	SEM	**Adj. R**^**2**^	**S**_**y|x**_	P (CFA)
**OXA-BMS (innovator)**	5.42	0.08	1.73	0.13	45.19	2.32	62.24	3.07	108.36	7.32	0.99	0.23	NA

**OXA-BLA**	3.38	0.10	3.29	0.79	19.38	2.65	**>750.00**	-	**>750.00**	-	0.98	0.21	<0.0001

**OXA-CAR**	3.69	0.10	1.81	0.20	25.44	2.20	97.61	16.13	**>750.00**	-	0.99	0.17	<0.0001

**OXA-COL**	3.14	0.12	1.93	0.36	35.41	3.71	132.29	33.04	**>750.00**	-	0.98	0.20	<0.0001

**OXA-MEM**	3.51	0.12	2.09	0.32	23.07	2.65	**>750.00**	-	**>750.00**	-	0.96	0.32	<0.0001

**OXA-OPH**	3.11	0.15	NS	-	NS	-	**>750.00**	-	**>750.00**	-	0.96	0.31	<0.0001*

**OXA-QIM**	2.22	0.10	2.72	0.60	25.00	3.95	**>750.00**	-	**>750.00**	-	0.96	0.20	<0.0001

**OXA-SCA**	3.66	0.17	2.03	0.46	19.31	3.02	**>750.00**	-	**>750.00**	-	0.95	0.31	<0.0001

**OXA-SER**†	-	-	-	-	-	-	-	-	-	-	-	-	NA

**OXA-VIT**	3.01	0.12	1.55	0.26	18.51	2.50	**>750.00**	-	**>750.00**	-	0.97	0.20	<0.0001

**Abbreviators**: E_max_: maximum effect; N: Hill's slope; ED_50_: effective dose to reach 50% of E_max_; BD: bacteriostatic dose; 1LKD: 1 log kill dose; Adj. R^2^: adjusted coefficient of determination; S_y|x_: standard error of the estimate; SEM: standard error of the mean; NA: not applicable; **Bold numbers**: not statistically different from zero, invalid parameter. * CFA comparison of E_max _only. † Pharmacodynamic profile fits to Gaussian instead of Hill's model.

Using Akaike's Information Criteria, we demonstrated a different pharmacodynamic pattern for OXA-SER, the only product in which greater doses killed fewer bacteria (Eagle effect). The probability of the model being correct was 92.5% for the Gaussian and 7.52% for the Hill model, therefore the former was adopted: E_max _= -5.1 log_10 _CFU/g, logEC_50 _= 1.78, EC_50 _= 60.3 mg/kg per day, slope = 0.87 (data shown in Table 4 includes only OXA products fitting Hill's model).

Table 5 shows results from multivariate nonlinear regression (M-NLR) under Hill's model (this precluded the use of OXA-SER). In order to fulfill the regression requirements of normality and homoscedasticity, each experiment was analyzed separately. As explained under Methods, fixing the E_max _to the value reached by OXA-BMS creates a hypothetical situation where all products are as effective as the innovator and allows calculation of their true potency in vivo. M-NLR demonstrated that generic products would have needed 3.33-15.2 and 2.38-8.48 greater doses to attain bacteriostasis (BD) and to kill the first log (1LKD), respectively. OXA-OPH generated invalid BD and 1LKD parameters (i.e., not statistically different from zero) due to extremely poor effects, and all but 3 generics (OXA-CAR, OXA-QIM and OXA-COL) generated invalid 1LKD.

**Table 5:** In vivo pharmacodynamic parameters of oxacillin products by multiple nonlinear regression (M-NLR).

OXACILLIN (OXA) PRODUCTS	N	SEM	**ED**_**50 **_**(mg/kg)**	SEM	BD (mg/kg)	SEM	1LKD (mg/kg)	SEM
**Experiment 1**								

OXA-BMS (innovator)	1.73	0.30	45.13	5.47	77.73	0.20	104.79	24.18

OXA-OPH	0.47	0.09	95.15	35.54	**>750.00**	-	**>750.00**	-

OXA-MEM	0.56	0.10	76.42	24.90	409.36	182.35	**>750.00**	-

OXA-SCA	0.56	0.10	72.66	23.71	390.13	172.68	**>750.00**	-

OXA-VIT	0.45	0.10	205.11	84.98	750.00*	373.73	**>750.00**	-

**Experiment 2**								

OXA-BMS (innovator)	1.73	0.18	45.13	3.33	60.71	4.33	96.48	12.24

OXA-CAR	0.64	0.07	90.52	16.50	202.51	39.36	230.84	230.84

**Experiment 3**								

OXA-BMS (innovator)	1.73	0.29	45.13	5.39	62.17	7.07	97.35	20.07

OXA-MEM	0.63	0.12	127.53	38.29	307.47	101.82	**>750.00**	-

OXA-OPH	0.55	0.10	128.00	42.18	526.98	225.98	**>750.00**	-

OXA-BLA	0.59	0.11	101.00	31.58	259.22	88.45	**>750.00**	-

**Experiment 4**								

OXA-BMS (innovator)	1.73	0.21	45.13	3.96	49.23	3.83	88.41	17.53

OXA-QUIM	0.40	0.08	750.00	321.55	750.00*	287.80	750.00*	327.88

OXA-COL	0.58	0.09	221.34	54.59	287.58	70.52	750.00*	356.95

Under this analysis, maximum effect (E_max_) for all products is fixed to the innovator's (E_max_= 5.42 log_10 _CFU/g).

**Abbreviators**: N: Hill's slope; ED_50_: effective dose to reach 50% of E_max_; BD: bacteriostatic dose; 1LKD: 1 log kill dose; SEM: standard error of the mean; **Bold numbers**: not statistically different from zero, invalid parameter. * Maximum dose used in the experiments.

## Discussion

These data demonstrate that generic products of oxacillin lack therapeutic equivalence in an animal model, despite the fact that 5 of 9 displayed pharmaceutical equivalence in a validated microbiological assay. The other 4 products did differ in potency, manifested by the divergent slopes of their standard curves compared with the innovator. This finding suggests that the molecules were not identical to the comparator and that subtle alterations, due to possible structural differences, spatial orientation, and/or impurities, might have affected their antibacterial activity in the microbiological assay and, therefore, in vivo [13]. While therapeutic inequivalence of these 4 generics with inferior potency was expected and understandable, in vivo failure of the 5 products that demonstrated pharmaceutical equivalence contradicts current assumptions made by patients, physicians, DRA and the World Health Organization (WHO) [13-17].

All generics had lower ED_50 _than the innovator OXA, showing a seemingly greater potency. It is in fact apparent, because the PDP potency (ED_50_) is relative to the compound's efficacy (E_max_), which was 22 to 1585-fold lower than the innovator's. This difference in potency disappears when secondary PDP are computed. These parameters convey more biological sense to the clinician because they are based on the amount of drug needed to reach measurable bacteriostatic and bactericidal effects for the bacterial load and growth suffered by the animal during the experiment. As shown in Table 4, OXA-CAR and OXA-COL required 1.57 and 2.12-times greater amount of drug to reach the BD while it could not be calculated for the other products because either they failed to kill at least 3.4 log_10 _CFU/g of bacteria (average growth in controls) or did not fit to Hill's model (OXA-SER). Notably, no generic reached 1LKD (i.e. a reduction of at least 4.4 log_10 _CFU/g compared to controls). This fact is further demonstrated with the multiple NLR (Table 5), in which, after equating efficacy (E_max_), all generics require higher ED_50_, BD and 1LKD. It means that, assuming that generics were as effective as the innovator product, all are significantly less potent independently of the PDP used.

As explained in Methods, oxacillin products were purchased on demand and it was not possible to obtain identical batches of each product for both in vitro and in vivo testing. To make sure that failure was batch-independent, the design included at least 2 batches per product (if available), and so one batch was studied both in vitro **and **vivo, and the other(s) in vitro **or **in vivo (Table 1). As a result, we allocated the same batch of the innovator and 6 generics to MIC, MBC and in vivo experiments, and the same batch of the other 5 generics to microbiological assays and in vivo experiments. Since no product failed susceptibility tests (5 of 9 had the same batch studied in the animal model), 4 failed in microbiological assays (2 had the same batch studied in the animal model), and all generics failed in vivo (all had the same batch studied in vitro, 6 for MIC & MBC and the other 5 for microbiological assay), the design demonstrates that therapeutic inequivalence is not a batch-related but a product-related problem.

One more clarification is pertinent considering that readers would like to know about results with products excluded for technical reasons (OXA-EXP and OXA-PEN, animal model) or due to unavailability at the time of certain experiments (OXA-QIM and OXA-SER, microbiological assay; OXA-EXP and OXA-SCA, susceptibility testing). One experiment in the animal model had to be excluded because of contamination that precluded a reliable bacterial count in the plates. Data from that experiment involved three generic products (OXA-EXP, OXA-PEN, OXA-VIT) and the innovator (OXA-BMS). Although the data are not shown, it should be noted that all generics in that experiment were significantly inferior respect to the innovator (E_max _= 4.85 ± 0.28, 4.74 ± 0.53, 3.02 ± 0.20, and 6.20 ± 0.22, respectively; P < 0.0001 by CFA). Regarding unavailable products, no conclusion can be made about pharmaceutical equivalence of OXA-QIM and OXA-SER, but both failed in vivo (please see further discussion about the last product below). OXA-SCA lacked MIC and MBC data, its potency and concentration were undistinguishable from the innovator, but failed in vivo too. Results with the other products suggest that standard susceptibility testing cannot detect differences among pharmaceutically and/or therapeutically inequivalent generics, which is explained by the very low power of this in vitro method in comparison with the microbiological assay (7.1% versus 98.9%, calculated with Sigma Plot 10.0 and StudySize 2.0).

The Eagle effect displayed by OXA-SER explains its Gaussian pharmacodynamic pattern (Figure 3), described at the beginnings of the antibiotic era for penicillins and later for other antibacterials [18]. This case was unique among oxacillin generics; OXA-SER probably had degradation products in its formulation that competed with the API for the molecular target, as we described recently with generics of vancomycin [19].

The other 8 generics had pharmacodynamic profiles fitting to Hill's model, but with markedly inferior efficacy; although several reasons could potentially explain their failure, the fact that it was detected only in vivo suggests that these compounds change when their targets (bacteria) are located in tissues instead of culture media. Oxacillin is the most unstable isoxazolyl penicillin: it starts degrading as soon as entering the body, to the point that by 12 h half of the drug has been eliminated as penicilloic acid in healthy subjects [20]. Instability has already been reported as the cause of therapeutic failure with a generic product of the betalactam cefuroxime [21]. There is evidence that oxacillin's resistance to bacterial beta-lactamases depends on the relative rigidity of the 6 β-side chain and the nature and orientation of substituents beyond the 6 β-amide carbonyl group [22]. Biochemical conditions affect these molecular interactions, and many biodynamic variables occur in the live animal that are absent in vitro, particularly in standardized tests like those employed to detect pharmaceutical equivalence or susceptibility to antimicrobials. The lack of one or both of the above mentioned characteristics in the site of infection (caused by an unstable API or by less-than-ideal excipients) could also generate a poor target for PBP2, explaining in vivo failure of generic oxacillin. To test this hypothesis, one could compare the spatial coordinates of generic products with those of the innovator, as well as their crystal structures and conformational energy maps in culture media and in infected tissues [23].

We found no studies regarding generic products of oxacillin. It is not surprising because (1) nobody has looked so far into therapeutic equivalence of generic medicines in general, and (2) the innovator of oxacillin abandoned its product several years ago. An entire society (like Colombian People) without therapeutically equivalent oxacillin is a highly dangerous consequence derived from the usual strategy taken by innovator pharmaceutical industry (to abandon the product) to evade the insurmountable competition represented by inexpensive, often ludicrously cheap generic products. The interest of big pharma in creating marketing niches for their new, very expensive compounds (free of generic competition) is generating a second consequence involving many old (but critically important) antibiotics. The industry must demonstrate to DRA that their new compounds have any advantage at all compared with the old, reliable, inexpensive antimicrobials. If one looks carefully at these campaigns, virtually all clinical trials compare the new compound against well-established drugs such as vancomycin or oxacillin, but without identifying their maker. It is not uncommon for the new drug to end the winner, but one wonders how many of these research subjects were treated with the innovator of vancomycin or oxacillin, and how many with inequivalent generics from multiple sources.

The consequences of prescribing generic oxacillin devoid of therapeutic equivalence are indeed serious, and affect more people than the sick individual receiving the drug. Patients with non-lethal but serious staphylococcal infections would take longer to get better, increasing the costs of their care and the probability of nosocomial infections or sequelae from the poorly treated staphylococcal infection. Those with life-threatening infections are at risk of dying due to ineffective therapy, but it might pass unnoticed by the physician, who could attribute death to an inevitable bad prognosis. One million bugs per gram of tissue left alive by the failing generic could increase its chances of selecting resistant mutants, making resistance a theoretical possibility [24]. It would be the most concerning consequence of massive use of pharmaceutically equivalent but therapeutically inequivalent generic antibiotics. Our current research points to that direction [25].

## Conclusions

Pharmaceutical equivalence or similar MIC & MBC values of any generic product of oxacillin are not useful criteria for granting therapeutic equivalence. In order to do it, all generic products of oxacillin should be tested in vivo. Otherwise, clinicians would expose patients to an unacceptable risk of treatment failure, especially immunosuppressed and critically ill patients who rely almost completely on antibiotic efficacy for cure. The WHO and DRA should think again this delicate issue; thorough demonstration of therapeutic equivalence must be required from generic makers as the case of oxacillin is not unique [26]. Serious commitment to improve global health, demonstrated by keeping their support for old and inexpensive antimicrobials like oxacillin, would be the minimum to ask from innovators.

## Abbreviations

1LKD: One-log kill dose; API: Active Pharmaceutical Ingredient; BD: Bacteriostatic Dose; CFA: Curve Fitting Analysis; E_max_: Maximum Effect; ED_50_: effective dose to reach 50% of the E_max_; N: slope; MIC: Minimal Inhibitory Concentration; MBC: Minimal Bactericidal Concentration; SPF: specific pathogen-free; MRSA: Methicillin-Resistant *Staphylococcus aureus*; N: Slope; NLR: Non-Linear Regression; OXA: Oxacillin; PDP: Pharmacodynamic parameter; PK/PD: Pharmacokinetics and Pharmacodynamics; SEM: Standard error of the mean; S_y|x_: Standard Error of the Estimate.

## Competing interests

C.A. Rodriguez and M. Agudelo have received financial help to participate in international meetings from AztraZeneca and Wyeth. A.F. Zuluaga has received honoraria for lectures from Pfizer, Allergan and Roche, and financial support from Merck Sharp & Dohme (MSD) to participate in international meetings. O. Vesga has received honoraria for lectures and financial support to participate in international meetings from GlaxoSmithKline, Bristol-Myers Squibb (BMS), AstraZeneca, MSD, and Wyeth, and has been a member of advisory boards for Wyeth.

## Authors' contributions

CAR, MA and AFZ performed the experiments, participated in the analysis of data and the preparation of the manuscript. OV designed and supervised the whole project, revised and analyzed the data. All authors read and approved the final manuscript.

## Pre-publication history

The pre-publication history for this paper can be accessed here:

http://www.biomedcentral.com/1471-2334/10/153/prepub
